# KRAS Promotes GLI2-Dependent Transcription during Pancreatic Carcinogenesis

**DOI:** 10.1158/2767-9764.CRC-23-0464

**Published:** 2024-07-09

**Authors:** Ashley N. Sigafoos, Ezequiel J. Tolosa, Ryan M. Carr, Maite G. Fernandez-Barrena, Luciana L. Almada, David R. Pease, Tara L. Hogenson, Glancis L. Raja Arul, Fatemeh Mousavi, Sandhya Sen, Renzo E. Vera, David L. Marks, Luis F. Flores, Kayla C. LaRue-Nolan, Chen Wu, William R. Bamlet, Anne M. Vrabel, Hugues Sicotte, Erin L. Schenk, Thomas C. Smyrk, Lizhi Zhang, Kari G. Rabe, Ann L. Oberg, Peter G. Zaphiropoulos, Eric Chevet, Rondell P. Graham, Catherine E. Hagen, Marina P. di Magliano, Sherine F. Elsawa, Christopher L. Pin, Junhao Mao, Robert R. McWilliams, Martin E. Fernandez-Zapico

**Affiliations:** 1 Division of Oncology Research, Schulze Center for Novel Therapeutics, Mayo Clinic, Rochester, Minnesota.; 2 Division of Medical Oncology, Mayo Clinic, Rochester, Minnesota.; 3 Department of Physiology and Pharmacology, University of Western Ontario, London, Canada.; 4 Department of Oncology, University of Western Ontario, London, Canada.; 5 Department of Health Sciences Research, Mayo Clinic, Rochester, Minnesota.; 6 Department of Biosciences and Nutrition, Karolinska Institutet, Huddinge, Sweden.; 7 Université de Rennes, CEDEX, Rennes, France.; 8 Division of Anatomic Pathology, Mayo Clinic, Rochester, Minnesota.; 9 Cellular and Molecular Biology Program, University of Michigan, Ann Arbor, Michigan.; 10 Department of Molecular, Cellular and Biomedical Sciences, University of New Hampshire, Durham, New Hampshire.; 11 University of Massachusetts Medical School, Worcester, Massachusetts.

## Abstract

Aberrant activation of GLI transcription factors has been implicated in the pathogenesis of different tumor types including pancreatic ductal adenocarcinoma. However, the mechanistic link with established drivers of this disease remains in part elusive. In this study, using a new genetically engineered mouse model overexpressing constitutively active mouse form of GLI2 and a combination of genome-wide assays, we provide evidence of a novel mechanism underlying the interplay between *KRAS*, a major driver of pancreatic ductal adenocarcinoma development, and *GLI2* to control oncogenic gene expression. These mice, also expressing *Kras*^*G12D*^, show significantly reduced median survival rate and accelerated tumorigenesis compared with the *Kras*^*G12D*^ only expressing mice. Analysis of the mechanism using RNA sequencing demonstrate higher levels of GLI2 targets, particularly tumor growth–promoting genes, including *Ccnd1*, *N-Myc*, and *Bcl2*, in *Kras*^*G12D*^ mutant cells. Furthermore, chromatin immunoprecipitation sequencing studies showed that in these cells *Kras*^*G12D*^ increases the levels of trimethylation of lysine 4 of the histone 3 (H3K4me3) at the promoter of GLI2 targets without affecting significantly the levels of other major active chromatin marks. Importantly, Gli2 knockdown reduces H3K4me3 enrichment and gene expression induced by mutant *Kras*. In summary, we demonstrate that Gli2 plays a significant role in pancreatic carcinogenesis by acting as a downstream effector of *Kras*^*G12D*^ to control gene expression.

## Introduction

The GLI family of zinc finger transcription factors (TF) are a part of the Hedgehog (Hh) signaling pathway and control transcriptional programs mediating pathway activation (1). This highly conserved TF family was first identified by Nusslein-Volhard and Wieschaus in *Drosophila melanogaster* (2). Vertebrates have three distinct GLI genes: *GLI1*, *GLI2*, and *GLI3*, each of which mediates complex biological processes. GLI proteins interact with one another to form a context and tissue-dependent GLI code that facilitates complex functions during early development, including cell proliferation, differentiation, and survival (3, 4). Increased GLI expression and activity has been associated with poor prognosis, development of high-grade tumors and resistance to chemotherapy in multiple malignancies (5, 6) including pancreatic ductal adenocarcinoma (PDAC; refs. 7–13). Ligand-dependent and -independent aberrant activation of the Hh/GLI signaling pathway has been observed in PDAC initiation and progression (7–13). Interestingly, this activation takes place in different cellular compartments within the tumor driving distinct features of PDAC biology (14–18).

Given the established correlation between aberrant Hh/GLI pathway activation and PDAC pathogenesis, we sought to investigate the effects of genetic variation identified in Hh pathway components among patients with PDAC and uncover any potential correlation to clinical outcomes. We identified a single-nucleotide polymorphism (SNP) in *GLI2* through PDAC genome-wide association studies (GWAS) that correlated with increased *GLI2* expression and poor patient survival. Using a novel PDAC mouse model expressing oncogenic *Kras* (*Kras*^*G12D*^) and constitutively active *Gli2*, we demonstrated an increased incidence of poorly differentiated adenocarcinoma and decreased survival. To understand the mechanism, we performed RNA sequencing (RNA-seq) analysis in murine cell lines expressing oncogenic mutant *Kras*^*G12D*^, which further revealed upregulation of both *Gli2* and GLI target genes including *Ccnd1*, *N-Myc*, and *Bcl2*. Analysis of active chromatin marks using chromatin immunoprecipitation sequencing (ChIP-seq) assays, found to have significant enrichment of the trimethylation of lysine 4 of the histone 3 (H3K4me3) at GLI target genes. Using Ccnd1 as a model, we demonstrated GLI2 occupancy was confirmed at target promoter, and knockdown of GLI2 in *Kras*^*G12D*^-expressing cells reduced H3K4me3 enrichment and target gene expression. In summary, we demonstrate that GLI2 plays a significant role in pancreatic carcinogenesis by acting as a downstream effector of KRAS, upregulating gene expression, and increasing H3K4me3 enrichment of GLI target genes.

## Materials and Methods

### Study design

This study was composed of a two-stage design, utilizing cases from the Mayo Clinic, recruited from October 1, 2000, to July 1, 2010 (Supplementary Table S1). The first stage was composed of 590 patients with PDAC of all stages included in a GWAS (19, 20). DNA samples for GWAS were collected from blood or buccal cells from patients diagnosed with primary adenocarcinoma of the exocrine pancreas. The second stage was composed of an additional 905 patients with PDAC from the same source, who were subsequently genotyped for polymorphisms identified with statistically significant survival associations (*P* < 0.05) in the first stage. For the second stage, paraffin-embedded sections (4 µm thickness) of human PDACs were analyzed as described previously (21). Recruitment, consent, and biospecimen collection were approved by our institutional review board and have been described previously (20). The study was conducted according to the Declaration of Helsinki, the Good Clinical Practice guidelines of the International Conference on Harmonization. Patient records were reviewed by a subspecialist in gastrointestinal oncology and confirmed as PDAC. Ninety-six percent had histologic confirmation of their PDAC by two expert pathologists with the remainder meeting prespecified criteria for a clinical diagnosis of PDAC. All patients completed a risk factor questionnaire at enrollment including self-reported Karnofsky performance score, lifestyle, and family history information. Staging was performed at study entry using American Joint Committee on Cancer sixth edition criteria. For analysis purposes, patients were identified as resected, locally advanced, or metastatic. Resected patients were further categorized as stage IA, IB, IIA, or IIB for the survival analyses shown in Supplementary Table S1. As Mayo Clinic is a referral center and there may be some delay from initial diagnosis or suspected diagnosis to presentation at our center, we performed sensitivity analyses using the date the diagnosis was first suspected as the starting point for survival analyses.

### Survival association analysis

Survival time was defined as the time elapsed from the date of the first visit to Mayo Clinic for pancreatic cancer diagnosis confirmation to date of death. To minimize lead time bias, patients were excluded if their first visit to our center was more than 3 months after their initial diagnosis of cancer. Date of death was obtained from online sources (Accurint^®^), death certificates, medical records, or family communication. Survival analyses were performed using a Cox proportional hazard regression model from date of diagnosis at our center to death or last follow-up, adjusted for clinical stage, age, sex, and Karnofsky performance score. Treatment data were not included in the model as available treatments for the time period of the study have minimal effect on survival. FOLFIRINOX, a triple combination of irinotecan, oxaliplatin, and 5-flurouracil that has been associated with notably improved survival (22), was not in widespread use in our patient population during this time (2000–2010). Genotype data were incorporated into the regression models based on an additive, dominant, recessive, or codominant genotype effect for the minor allele. Subset analysis of stage groupings (resected, locally advanced, or metastatic) was performed using an additive model to determine stage-specific effects (Supplementary Table S2).

### Genotyping

Genotyping for the first stage was performed as part of a GWAS using the Illumina 550K platform (*N* = 590) or Illumina 610K (*N* = 905) using methodology previously described (19, 20). In the second stage, 35 candidate SNPs were genotyped using Illumina 384-plex GoldenGate custom OPA methodology previously described (23).

### Cell lines

Human pancreatic cancer cell line Panc-1 were procured from ATCC and was cultured in DMEM (10-013-CV, Corning) supplemented with 10% heat-inactivated FBS (35-010-CV, Corning). Murine KC cell line was derived from primary tumor from our colony of *Ptf1a-****C****re;LSL-****K****ras*^G12D^ (KC; mouse ID: M2321) and cultured in DMEM + 10% FBS supplemented with 1× penicillin-streptomycin (15140122, Gibco). 1012U doxycycline-inducible cells were derived from a previously described genetically engineered mouse model (GEMM; ref. 24) and cultured in RPMI + 10% FBS (10-040-CV). Cells were maintained at 37°C and 5% CO_2_. Authentication of cell lines was done by genotyping as described by Collins and colleagues (24) and by copy-number variation analysis using BioPrime labeling kit (18094011, Invitrogen). All cell lines were tested for *Mycoplasma* using Universal Mycoplasma Detection Kit (30-1012K, ATCC) at the beginning and one additional time during the timepoints of the experimental design. All of them were negative during the length of our experiments. Between sample collections there was an average of 1 to 2 passages in all our studies.

### Expression analysis

Transcript expression in patient samples was determined qRT-PCR using TaqMan fluorescence methodology and ABI 7900 (Applied Biosystems). Predesigned primer/probe sets for *SHH* (HS01123832-M1), *GLI1* (HS01110766-M1), *GLI2* (HS01119974-M1), and *18s rRNA* (HS99999901-S1) expression were purchased from Applied Biosystems. RNA isolation was done following the protocol in the Qiagen RNeasy kit (74004). Five micrograms of RNA was reverse-transcribed using the High-Capacity cDNA Reverse Transcription Kit (4368814, Applied Biosystems). All reactions including controls were performed in triplicate. The relative target gene expression was normalized to the endogenous reference gene (*18s rRNA*) and determined using the ΔΔC_T_ method.

For the expression analysis from animal tissue, RNA from a 3-mm^3^ fresh or frozen piece of mouse pancreatic tissue was extracted with a PRO scientific homogenizer and TRIzol (15596026, Invitrogen) first and then, isolated using RNeasy kit according to the manufacturer’s instructions. RT reactions were performed using a High-Capacity cDNA RT kit and cDNA samples were used for qPCR with SsoAdvanced SYBR Green PCR Master Mix (1725274, Bio-Rad) and primers of interest (Supplementary Table S3). qPCR cycling conditions used were as follows: 95°C for 5 minutes followed by 40 cycles of 95°C for 15 seconds and 60°C for 1 minute. Target gene expression was normalized to reference genes (*Tbp* and *Prt*) and calculated using ΔΔC_T_ method. Expression analysis in cell lines was performed using TRIzol following the kit protocol. Two micrograms of total RNA was reverse-transcribed using a high-capacity cDNA RT kit. A portion of the total cDNA was amplified by qRT-PCR, using 1× IQ SYBR Green Supermix (1708880, Bio-Rad) and primers for target genes (Supplementary Table S3). Amplification was performed using the C1000 Thermal Cycler under the following reaction conditions: 95°C for 3 minutes followed by 40 cycles of 30 seconds at 95°C, 30 seconds at 60°C, and 20 seconds at 72°C. Each mRNA level was normalized by comparison with human/mouse *TBP*/*Tbp* and *PRT*/*Prt*. The results were calculated following the ΔΔC_T_ method.

### Breeding

Animals were housed in pathogen-free conditions in facilities reviewed and approved by the American Association for Accreditation of Laboratory Animal Care per current regulations and standards of the United States Department of Agriculture, Department of Health and Human Services, and NIH/Institutional Animal Care and Use Committee. *Ptf1a-Cre* and *LSL-Kras*^G12D^ animals were previously described (21, 25, 26). To generate the *Rosa26*-*ΔNGli2* mouse, the cDNA fragment encoding the N-terminally truncated form of *Gli2* carrying a N-terminal Flag-tag and C-terminal IRES-nuclear *LacZ* was inserted in the *Rosa26* locus [detailed description in (27)]. *Ptf1a-Cre* and *Rosa26-ΔNGli2* mice were crossed to generate: (i) *Ptf1a-Cre* (Cre; ii) *Ptf1a-****C****re;****R****osa26 ΔN****G****li2* (CRG) mice. Progeny from this group was then crossed with *LSL-Kras*^G12D^ to generate: KC and *Ptf1a-****C****re;LSL-****K****ras*^G12D^;***R****osa26 ΔN****G****li2* (KCRG) mice. Cre and KC mice were used as controls in experiments. A minimum of 12 mice were aged for survival per cohort and Kaplan–Meier curves generated to evaluate the effect of the active form of *Gli2* expression in control and PDAC transgenic mouse models.

### *In vivo* genotyping

Tail DNA was isolated using 180 µL of 50 mmol/L NaOH with proteinase K, then incubated at 37°C for 30 minutes, followed by 95°C for 10 minutes. Twenty microliters of 1 mol/L Tris-EDTA (pH 7.4) was added to the samples following which, 2 µL of crude DNA was used for PCR amplification with Terra PCR Direct Red Dy Premix (639286, TaKaRa). The following genes were confirmed to be present: *Ptf1a-Cre*, *Kras*^G12D^, *Rosa26 ΔNGli2* (Supplementary Table S3). PCR cycling conditions used were as follows: 95°C for 5 minutes, 95°C for 30 seconds, 58°C or 63°C for 45 seconds, and 72°C for 1 minute for 34 cycles. Amplified PCR products were resolved on 1% to 2% agarose gels.

### IHC

IHC staining for phospho-ERK (p-ERK) was performed at the Pathology Research Core (Mayo Clinic) using the Leica Bond RX stainer (Leica). Formalin-fixed, paraffin-embedded tissues were sectioned at 5 microns and IHC staining was performed online. Slides for p-ERK 1/2 stain were retrieved for 20 minutes using BOND Epitope Retrieval Solution 1 (AR9961, Leica). The p-ERK 1/2 primary antibody (4370, Cell Signaling Technology) is a rabbit monoclonal and it was diluted to 1:1,200 in Background Reducing Diluent (S2022, Agilent Dako) and incubated for 15 minutes. The detection system used was Polymer Refine Detection System (Leica). This system includes the hydrogen peroxidase block, polymer reagent, 3,3′-diaminobenzidine (DAB), and hematoxylin. Immunostaining visualization was achieved by incubating slides 10 minutes in DAB and DAB buffer (1:19 mixture) from the Bond Polymer Refine Detection System. To this point, slides were rinsed between steps with 1× bond wash buffer (Leica). Slides were counterstained for 5 minutes using Schmidt hematoxylin and molecular biology grade water (1:1 mixture), followed by several rinses in 1× bond wash buffer and distilled water. Once the immunochemistry process was completed, slides were removed from the stainer and rinsed in tap water for 5 minutes. Slides were dehydrated in increasing concentrations of ethyl alcohol and cleared in three changes of xylene prior to permanent coverslipping in xylene-based medium.

IHC staining for Ki67, F4/80, CD4, and CD8 was performed in all four genotypes (Cre, CRG, KC, and KCRG). Slides were heated at 40°C for 30 minutes then rehydrated and run through xylene to remove the paraffin. Optimization was done for each antibody with the following conditions and concentrations used; antigen retrieval using 10 mmol/L sodium citrate buffer was used for Ki67 1:500 (ab66155, Abcam) and F4/80 1:200 (70076 S, Cell Signaling Technology). Antigen retrieval using 1 mmol/L EDTA pH 8 was used for CD4 1:100 (25229 S, Cell Signaling Technology) and CD8 1:200 (98941 S, Cell Signaling Technology). IHC application solution kit was used (13079, Cell Signaling Technology) and kit protocol was followed. Analysis of Ki67 IHC slides were performed using QuPath software for positive cell detection (28). Minimum of six independent fields per mice were captured and analyzed.

#### Histopathologic analysis and immunocytochemistry

Pancreas and spleen were collected from the mice and fixed in 10% neutral buffered formalin (427-098, Fisher Healthcare). Samples were processed by Mayo Clinic Histology Core Laboratory for paraffin embedding and sectioning for hematoxylin and eosin (H&E) and Masson trichrome staining. For staining, tissue slides were baked and run through xylene to remove paraffin. Tissue was then rehydrated and stained with hematoxylin and blued after which they were counterstained with eosin. For Masson trichrome, slides were stained with hematoxylin, Biebrich scarlet-acid fuchsin, and lastly counterstained with aniline blue. Tissues were then dehydrated, cleared, and mounted with mounting media. H&E tissue samples were blindly evaluated by histopathologists R.P. Graham and C.E. Hagen. Masson trichrome staining was analyzed using QuPath software to measure amount of collagen present (28).

### Plasmid constructs

*pCEFL3xHAmGli2* was obtained from Addgene (plasmid, #37671). Gibson assembly (E2611S, NEB) was used to make *pCEFL3xHA-∆NmGli2* and *pCEFL3xHA* (empty vector) plasmids. *pCEFL3xHA-∆NmGli2*: *pCEFL3xHAmGli2* was digested with *SpeI* and *BglII* to remove the N-terminus of mGLI2. Fragment was amplified using Gib-mGli2-F1/Gib-mGli2-R1 primers (Supplementary Table S3) and the 91 bp amplicon was gel purified. Vector and insert were then cloned together via Gibson Assembly protocol. Final *∆NGli2* plasmid contained amino acids A280-*1545. *pCEFL3xHA: pCEFL3xHAmGli2* was digested with *HindIII* and *NotI* to remove the m*Gli2*. Fragment was amplified using Gib-pCEFL-EV-F1/Gib- pCEFL-EV-R1 primers (Supplementary Table S3) and the 156 bp amplicon was gel purified. Vector and insert were then cloned together via Gibson Assembly protocol.

### Luciferase reporter assay

Panc-1, 1012U, and KC cell lines were plated in triplicate, at 1.5 × 10^5^ cells/well in six-well plates. *pGL3-Basic* reporter containing eight consecutive GLI binding sites upstream of the luciferase gene (8xGLI, gift from Dr. Chi-Chung Hui, University of Toronto) was co-transfected with one of the following plasmids for each cell line, *pCEFL3xHA* (empty vector), *pCEFL3xHAmGli2*, and *pCEFL3xHA-∆NmGli2*. Cells were transfected using Lipofectamine (18324012, Invitrogen) following the manufacturer’s protocol. After 24 to 48 hours of transfection, samples were harvested and prepared for luciferase assay using manufacturer’s protocol (E1501, Promega). Total protein was quantified using the Bio-Rad protein assay per manufacturer’s protocol (5000201, Bio-Rad). Luciferase signal was normalized to total protein to account for any variation among samples and then normalized to their respective experimental control.

### Western blot

After treatments, cells were harvested and lysed on ice for 15 minutes using RIPA buffer (50 mmol/L Tris, pH 7.5, 150 mmol/L NaCl, 5 mmol/L EDTA, 0.5% sodium deoxycholate, 1% NP-40, 0.1% SDS, and 1 mmol/L PMSF), and complete protease inhibitor cocktail (11697498001, Roche), sonicated, and cleared of debris by centrifugation. Cleared lysates were collected and total protein was quantified via BCA (23225, Thermo Fisher Scientific) and loaded (20–60 µg protein) in the appropriate gel (4%–20%, 4561094; 4%–15%, 4561086; 7.5%, 4561023, all from Bio-Rad). Proteins were resolved via SDS-PAGE and transferred onto PVDF membranes (IPVH00010; Millipore). Membranes were blocked using either 5% nonfat dry milk (Apex Chemicals and Reagents, 20-241) or 5% BSA (9048-46-8, RPI). The primary antibodies used were CCDN1 (1:500, SC-753, Santa Cruz Biotechnology), MAPK (ERK ½; 1:500, 9102S, Cell Signaling Technology), p-ERK ½ (1:500, mab18251, R&D Systems), vinculin (1:500, A302-535A, Bethyl Laboratories), monomethylation of the lysine 4 of the histone H3 (H3K4me1; 1:1,000, 5326s, Cell Signaling Technology), H3K4me3 (1:1,000, ab8580, Abcam), acetylation of the lysine 27 of the histone 3 (H3K27Ac; 1:1,000, ab4729, Abcam), and total H3 (1:1,000, 3638s, Cell Signaling Technology). CCND1 (1:1,000, 55506s, Cell Signaling Technology), HA (1:500, 42155800, Roche), and GLI2 (1:1,000, NB600-874, Novus Biologicals). The following secondary antibodies were used anti–rabbit IgG-HRP (1:2,000, 12-348, Millipore), anti–mouse IgG-HRP (1:2,000, AP124P, Millipore), and anti–rat IgG-HRP (1:2,000, AP136P, Millipore). Blots were developed using SuperSignal West Pico Chemiluminescent Substrate (34080, Thermo Fisher Scientific) and imaged using a Bio-Rad ChemiDoc Imaging System. Images were exported and viewed and quantify using ImageJ software.

### Chromatin immunoprecipitation

1012U cells were plated at 0.5 × 10^6^ in 15 cm dishes and treated with 1 µg/mL Dox every 24 hours and chromatin collected at 72 hours. DNA/proteins were cross-linked using 1% formaldehyde and then lysed. DNA was sheared using sonication (45 cycles of 30 seconds sonication followed by 30 seconds of rest; Diagenode Bioruptor 300). ChIP was performed as previously described (29), using 1 µg of normal rabbit IgG (2729s, Cell Signaling Technology) and rabbit anti-GLI2 (NB600-874, Novus Biologicals). Primers were used to amplify the promoter region of *Ccnd1* (Supplementary Table S3). qPCR was performed on the samples using SsoAdvanced SYBR Green PCR Master Mix.

For knockdown experiments, 1012U cells were plated at 0.5 × 10^6^ in 15 cm dishes and treated with 1 µg/mL doxycycline (fresh Dox added every 24 hours). For optimal Gli2 knockdown, cells were transfected twice, at 24 and 48 hours, with siRNA *Gli2* (1027418, GeneGlobe. SI02723637, Qiagen) or nontargeting control (NT) siRNA (1027418, GeneGlobe: SI03650318, Qiagen) using RNAiMax (13778150, Invitrogen) as per the manufacturer’s protocol. Cells were collected 48 hours post second transfection. DNA/protein were cross-linked using 1% formaldehyde and then lysed. DNA was sheared for 30 seconds sonication then 30 seconds of rest, repeating this cycle 45 times. ChIP was performed following a protocol previously described (29) using 1 µg normal rabbit IgG (2729s) or rabbit anti-H3K4me3 (04-745, Millipore Sigma) antibody. qPCR was performed using SsoAdvanced SYBR Green PCR Master Mix, targeting the Ccnd1 promoter region (Supplementary Table S3).

### RNA-seq analysis

For RNA-seq studies, 1012U cells were treated for 12, 24, 48, and 72 hours in RPMI media supplemented with 10% FBS and 1 µg/mL of Dox. RNA was extracted with TRIzol reagent and then isolated using RNeasy kit per manufacturer instructions. Library preparation and sequencing was outsourced to London Regional Genomics Centre at the University of Western Ontario. RNA-seq FASTq files were processed and trimmed with Trim Galore 0.6.4 and Cutadapt 2.10 with Python 3.6.5 (https://www.bioinformatics.babraham.ac.uk/projects/trim_galore/; ref. 30). Read quality was evaluated with FastQC (31) and aligned with STAR 2.7.3a and mm10 genome as reference using the Mayo Biocluster (32). The read counts and the differential gene expression were evaluated by FeatureCounts (Galaxy Version 1.6.4; (31) and DESeq2 (Galaxy Version 2.11.40.6; (33). Genes with a base mean ≥1, log_2_ fold change >1 or <−1, and a FDR of ≤0.05 were considered significantly differentially expressed. Heatmaps were generated in RStudio software using pheatmap version 1.0.12 (https://CRAN.R-project.org/package=pheatmap), and volcano plots were generated using EnhancedVolcano.

### Native ChIP-seq analysis

For native ChIP (N-ChIP), 1012U cells (4.5 × 10^6^) were resuspended in native lysis buffer (0.1% Triton X-100, 0.1% deoxycholate, and 1× protease inhibitor cocktail) and lysed on ice for 20 minutes. Shearing of the extracted chromatin was performed by incubating samples with 2.5 U/mL of MNase (M0247S, NEB) for 10 minutes at 25°C, followed by the addition of 250 µmol/L of EDTA to quench the reaction. A mix of 1% Triton X-100 and 1% deoxycholate was added to digested samples and incubated on ice for 20 minutes. Digested chromatin was precleared in IP buffer [20 mmol/L Tris-HCl (pH 7.5), 2 mmol/L EDTA, 150 mmol/L NaCl, 0.1% Triton X-100, 0.1% deoxycholate, and protease inhibitor cocktail] with prewashed protein G magnetic beads (10-004-D, Dynabeads, Thermo Fisher Scientific) for 1 hour at 4°C. Supernatants were aliquoted (0.5 × 10^6^ cells) and incubated with the corresponding antibody-bead complex (20 µL prewashed beads incubated for 1 hour at 4°C with 1 µg of antibody in IP buffer). Antibodies used were as follows: H3K4me3: Millipore; 07-449, H3K27Ac: Abcam; #ab4729, H3K4me1: Diagenode; #C15410037. Following overnight 4°C incubation, samples were washed twice with low salt buffer [20 mmol/L Tris-HCl (pH 8.0), 0.1% SDS, 1% Triton X-100, 2 mmol/L EDTA, and 150 mmol/L NaCl) and 2× with High Salt Buffer [20 mmol/L Tris-HCl (pH 8.0), 0.1% SDS, 1% Triton X-100, 2 mmol/L EDTA, and 500 mmol/L NaCl]. DNA–antibody complexes were eluted in elution buffer (100 mmol/L NaHCO3, 1% SDS), incubated at 65°C for 90 minutes. Protein digestion was performed on the eluted DNA samples at 50°C for 30 minutes (mix of EB buffer, G2 buffer, and Protease, all from Qiagen). ChIP DNA was purified using spin columns (IB47030, IBI Scientific). Input represents 1% of total sample. Library generation and sequencing was outsourced to the University of Western Ontario.

N-ChIP-seq FASTq files were aligned to mouse genome (mm10) using Bowtie2 (34). Significant peaks were called with MACS2 callpeak (35, 36). Differential binding analysis of called peaks was conducted with DiffBind with peaks summits set at 500 (37). Peaks with an FDR of ≤0.05 were considered significantly differentially bound. Overlapping H3K27Ac, H3K4me3, and H3K4me1 called peaks were identified using bedtools Intersect Intervals (38). Genes associated with overlapping H3K27Ac, H3K4me3, and H3K4me1 peaks were identified using GREAT analysis for single nearest gene within 1,000 kb (39, 40).

### Statistical analysis

Experimental data were first checked for normality and lognormality. Statistical significance between two groups was determined using unpaired Student *t* test or one-way ANOVA for three or more groups. A Dunnett test was employed as the *post hoc* test to determine statistical difference with respect to the control group. For nonparametric data, Kruskal–Wallis test was performed followed by a Dunn test to analyze the significance among groups. For Kaplan–Meier curves, statistical significance was calculated using log-rank (Mantel–Cox) analysis and a *P* < 0.05 was designated as statistically significant. Statistical analyses and corresponding graphical representation were done using GraphPad Prism 9 software. All data are expressed as mean ± SEM of at least three independent experiments, and *P* < 0.05 was considered statistically significant.

### Data availability

All data for this study is included in this version of the manuscript. Raw data from RNA-seq (BioProject ID PRJNA1112965) and ChIP-seq (BioProject ID PRJNA1113127) analyses have been deposited in the NCBI Sequence Read Archive data repository.

## Results

### GLI2 genetic defects are associated with its increased expression in human PDAC

Using a combination GWAS, we performed a two-stage survival association study in 1,495 patients with pancreatic cancer, correlating findings with tissue expression and histopathological analysis (Supplementary Table S1; refs. 19, 20). The data from these patients were used to construct a survival analysis study that focused specifically on 425 polymorphisms in the Hh pathway genes (*SHH*, *GLI1*, *GLI2*, *GLI3*, *SMO*, *SUFU*, *PTCH1*, *PTCH2*, and *FUSED*) available in the GWAS dataset. Twenty-three of the identified SNPs from stage 1 were statistically associated with differences in overall survival in an additive model adjusted for covariates of age, stage grouping, performance score, and usual adult body-mass index. These 23 SNPs were then assessed for correlation with survival in the second stage, which had a median survival of 277 days with 722 deaths (Supplementary Table S1). Only an intronic *GLI2* SNP rs1992901 was associated with lower overall survival in both stages (stage 1, HR, 1.14, *P* = 0.038; stage 2, HR, 1.21, *P* = 0.00068; Supplementary Table S2).

SNP may confer variations in gene expression through multiple mechanisms including through regulation of transcription (41), alterations in mRNA structure (42) affecting protein folding (43), or altering epitranscriptomic modifications (44).This SNP rs1992901 (NC_000002.12:g.120913093A>G on build 38) represents a change to adenosine from the ancestral guanine located in intron 1 of the *GLI2* gene on chromosome 2 (2q14). The allele frequency of the A-allele is 0.421 according to the 1000 genomes project. *In silico*, the minimum free energy (MFE) of the RNA segment flanking the SNP was calculated using the MFOLD program. This analysis revealed that *GLI2* wild-type (WT) MFE was 1,181.94 kcal/mol whereas the *GLI2* rs1992901 genotype was 1,552.03 kcal/mol suggesting higher stability. In addition, splicing prediction analysis demonstrates SNP rs1992901 generates a splice acceptor site, which is absent in the WT gene. Consequently, rs1992901 may preferentially result in a more energetically stable RNA splice variant and ultimately greater GLI2 expression. In fact, gene expression analysis performed in patient samples showed a statistically significant increase in *GLI2* expression in the homozygous and heterozygous *GLI2* SNP groups compared with the WT group (Supplementary Fig. S1A and S1B). Thus, the identified *GLI2* SNP rs1992901, which correlated with poor patient survival, was associated with increased *GLI2* expression in samples of patients with PDAC.

### Gli2 overexpression promotes Kras^G12D^-driven PDAC progression *in vivo*

On the basis of the above findings, we sought to assess the effect of increased GLI2 expression in the development of PDAC. To that end, we generated a GEMM CRG by crossing *Ptf1a-Cre* (Cre) mice with *Rosa26 ∆NGli2* (RG) mice, resulting in pancreas-specific expression of *∆NGli2* by *Ptf1a*-driven *Cre* (Supplementary Fig. S2A). Recombination of *Rosa26 ∆NGli2* in CRG mice was confirmed through qPCRs to assess *Gli2* and *LacZ* expression in mouse pancreas tissue (Supplementary Fig. S3A). This model expresses pancreas-specific GLI2 lacking the N-terminal repressor domain, resulting in constitutively active GLI2. Cre mice were used as controls for subsequent experiments. The increased transcriptional activity of ∆*NGli2* was confirmed in human PDAC line Panc-1 and two murine lines, one of them expressing doxycycline-induced oncogenic *Kras*^*G12D*^ (1012U +Dox), and the other expressing constitutively active *Kras*^*G12D*^ (KC) transfected a with WT *Gli2* or *∆NGli2*-expressing constructs. All experimental groups were co-transfected with an 8xGLI luciferase reporter construct containing eight consecutive GLI binding sites. In all lines, the overexpression of *∆NGli2* increased GLI transcriptional activity compared with both WT *Gli2*-expressing and control cells (Supplementary Fig. S3B). Western blots confirm protein expression of the WT *Gli2* and *∆NGli2* constructs showing that the increased luciferase activity of *∆NGli2* was not due to higher levels *∆NGli2* over WT *Gli2* (Supplementary Fig. S3C).

Phenotypic analysis showed no statistically significant differences in overall survival between the Cre and CRG mice (Supplementary Fig. S2B). We performed H&E staining on mice pancreas tissues from both cohorts to assess histological abnormalities, and the analysis showed no significant pathological alterations in CRG mice pancreas (Supplementary Fig. S2C, left). We further assessed the endocrine and exocrine compartments by using immunofluorescence to visualize insulin, glucagon, and amylase levels (Supplementary Fig. S2C, right) in mouse tissue sections. As observed with the H&E stain, we noted no significant alterations in the pancreas compartments.

**Figure 1:**
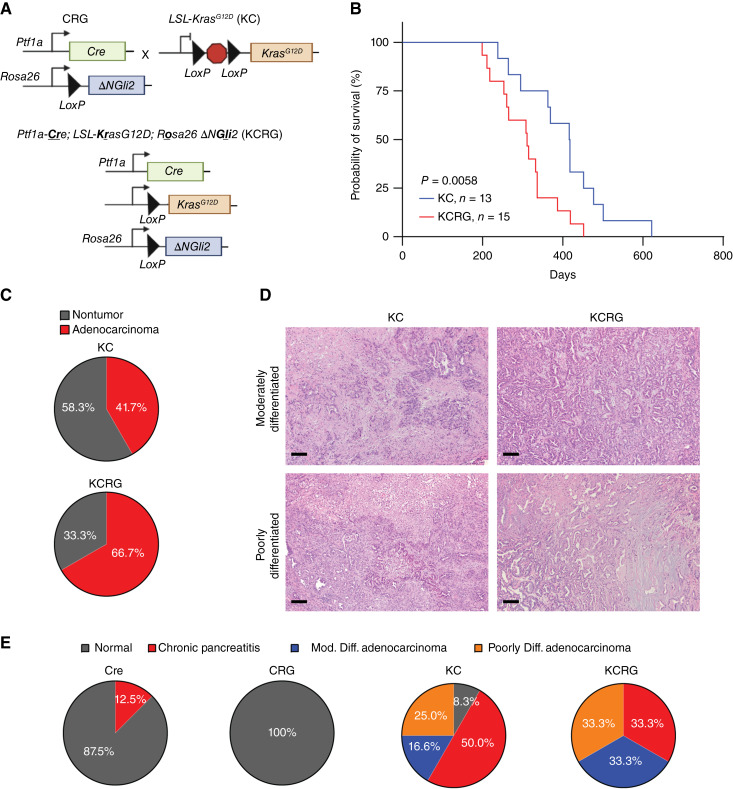
Gli2 promotes Kras-driven PDAC progression *in vivo.***A,** Representation of animal crosses to generate model KCRG mice using CRG and KC mice. **B,** Kaplan–Meier curve depicting survival in KC and KCRG animals (log-rank test, *P* = 0.0058). **C,** Pie charts representing percentage of nontumor and adenocarcinoma incidence in KC (*n* = 13) and KCRG (*n* = 15) mice. **D,** Representative H&E stain of KC and KCRG mice depicting moderately differentiated and poorly differentiated adenocarcinoma. Scale bar, 100 μm. **E,** Pie charts representing percentage incidence of chronic pancreatitis, moderately differentiated adenocarcinoma, and poorly differentiated adenocarcinoma in Cre (*n* = 9), CRG (*n* = 7), KC (*n* = 13), and KCRG (*n* = 15) animals.

Next, we sought to further assess the effect of increased GLI2 expression in a PDAC model system driven by mutant *Kras*^*G12D*^. CRG mice were crossed with *LSL*-*Kras*^G12D^ (KC) mice to generate KCRG. This model was used to investigate the outcomes of increased GLI2 expression in conjunction with KRAS^G12D^ expression (Fig. 1A). KC and Cre mice were used as experimental controls for subsequent experiments. The recombination of *Rosa26 ∆NGli2* in KCRG mice was validated through qPCR for *∆NGli2* and *LacZ* expression (Supplementary Fig. S3A). *∆NGli2* expression was significantly higher in CRG and KCRG mice compared with control Cre and KC mice respectively (Supplementary Fig. S3A, left). Similarly, *LacZ* expression was also significantly higher in KCRG mice compared with their respective controls (Supplementary Fig. S3A, right), indicative of successful recombination of *Rosa26 ∆NGli2* in KCRG mice. We further performed iIHC for p-ERK in the pancreas tissue of CRG, KC, and KCRG to verify KRAS activation. The results from the IHC show increased levels of p-ERK in KC and KCRG tissues, demonstrating KRAS activation, which is not observed in the CRG cohort in which mutant KRAS expression was not induced (Supplementary Fig. S4A).

The expression of both ∆NGLI2 and KRAS^G12D^ significantly reduced overall survival in the KCRG animals compared with the control KC animals that express only KRAS^G12D^ (*P* = 0.0058; Fig. 1B). Histopathology analyses performed in the two cohorts further show increased incidence of adenocarcinoma in the KCRG group compared with the KC group, as determined from quantification of tumor penetrance in mice belonging to both genotypes (Fig. 1C). Representative H&E images further depict moderately and poorly differentiated adenocarcinoma in KC and KCRG pancreatic tissue (Fig. 1D). More than 50% of KC animals developed chronic pancreatitis, as depicted in the pie chart (Fig. 1E) and representative H&E stains (Supplementary Fig. S4B). A relatively minor proportion of animals in the KC cohort developed moderately differentiated (16.6%) and poorly differentiated (25%) adenocarcinoma (Fig. 1E). The observations in the KCRG cohort deviate from those in the KC cohort. In the KCRG cohort, we noted an equal distribution of animals developing chronic pancreatitis, moderately and poorly differentiated adenocarcinoma (33% each; Fig. 1E).

**Figure 2:**
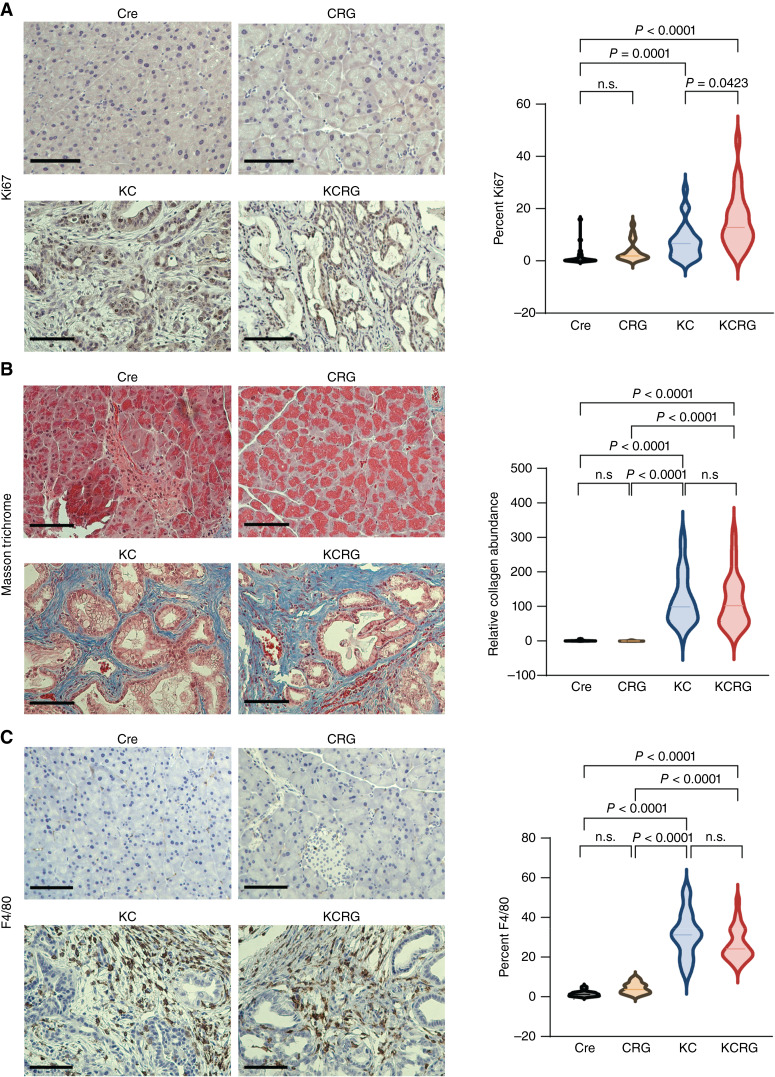
KCRG mice show increased tumor proliferation. **A,** Representative IHC images (left) and quantification (right) for the cell proliferation marker Ki67 (brown signal) in Cre (*n* = 3), CRG (*n* = 3), KC (*n* = 4), and KCRG (*n* = 4) mice. Scale bar, 200 μm. **B,** Masson trichrome staining immunocytochemistry representative images (left) and quantification (right) for collagen (blue signal) in Cre (*n* = 3) and CRG (*n* = 3) mice and KC (*n* = 4) and KCRG (*n* = 4) mice. Scale bar, 200 μm. **C,** Representative IHC images (left) and quantification (right) for macrophage marker F4/80 (brown signal) in Cre (*n* = 3), CRG (*n* = 3), KC (*n* = 4), and KCRG (*n* = 4) mice. Scale bar, 200 μm.

The level of proliferation in all genotypes was determined by IHC for Ki67. Interestingly, Ki67 levels showed a statistically significant increased level in KCRG mice compared with their control model KC animals with an average percent of Ki67^+^ cells (over total number of cells/field) of 15.5% and 7.5%, respectively (Fig. 2A). Next, slides were analyzed by two expert pathologists to determined tumor to stroma ratio (TSR). TSR was defined by the proportion of tumor cells relative to surrounding stroma and it was assigned one of two categories, amount of TSR <75% and amount of TSR >75%. Results showed that KC mice had a lower amount of TSR >75% when compared with KCRG, 60% and 80%, respectively. Masson Trichrome staining was performed to determine the relative collagen levels in pancreatic tissues. KC and KCRG show no differences but both of them have statistically significant amount of collagen compared with control Cre and CRG (Fig. 2B). Finally, IHC was performed to determine immune response. All four groups were stained for F4/80, CD4, and CD8. The immune landscape of these tumors in the KC versus KCRG groups seemed comparable for levels of F4/80 (Fig. 2C), CD4, and CD8 (Supplementary Fig. S5). Together, these results indicate the more aggressive nature of KCRG tumors and that this feature may be due to increased proliferative capacities of these malignancies.

### Oncogenic Kras induces GLI2 target gene expression

**Figure 3:**
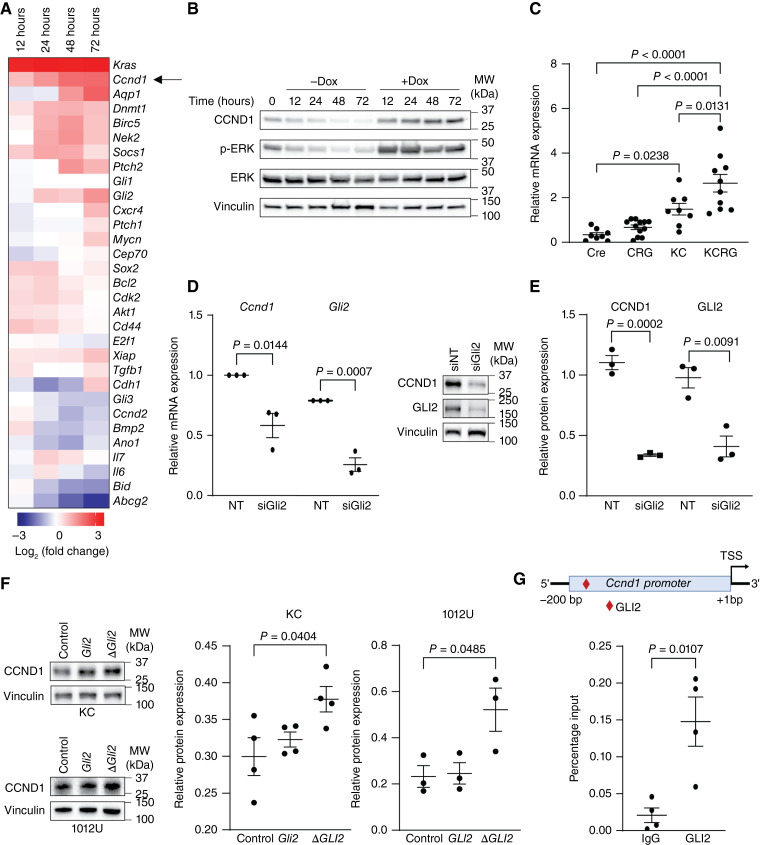
Gli2 is required for the regulation of *Ccnd1* downstream of oncogenic KRAS. **A,** Heatmap of GLI target gene expression in 1012U cells at 12, 24, 48, and 72 hours posttreatment with doxycycline (Dox). **B,** Relative gene expression of *Ccnd1* by qPCR in mouse pancreas tissue from Cre, CRG, KC, and KCRG mice. **C,** Western blot showing expression of p-ERK, total ERK, and CCND1 in 1012U −Dox and +Dox cells at 12, 24, 48, and 72 hours post treatment. Vinculin is used as the loading control. **D,** Relative gene expression of *Ccnd1* and *Gli2* by qPCR after the knockdown of *Gli2* by siRNA in 1012U cells +Dox cells (72 hours treatment; *P* < 0.05 and *P* < 0.001). **E,** Western blot (left) and protein quantification (right) representing expression of CCDN1 and GLI2 in 1012U cells +Dox, after the knockdown of *Gli2*. Vinculin is used as the loading control. **F,** Western blot (left) and protein quantification (right) representing expression of CCDN1 and GLI2 in 1012U cells +Dox and murine KC cell line transfected with *Gli2*, *∆NGli2*, or empty vector (control). Vinculin is used as the loading control. **G,** Top: Graphical representative of mouse *Ccnd1* promoter region with (red) diamond indicating candidate Gli2-binding site. Bottom: Graph representing enrichment (percent input) of GLI2 at the *Ccnd1* promoter in 1012U +Dox cells (72 hours treatment; *P* < 0.05).

To define the mechanism underlying the interplay between oncogenic *Kras* and GLI2, RNA-seq analysis was performed in our *Kras*-inducible 1012U cells at 12, 24, 48, and 72 hours post-doxycycline treatment (Supplementary Fig. S6A). Our global analysis showed a progressive increase in differentially expressed genes from 12 to 72 hours (Supplementary Fig. S6B). In particular, we noted an upregulation of several previously defined GLI target genes (45) with the induction of mutant KRAS expression (+Dox) compared with the not induced condition (−Dox; Fig. 3A, Supplementary Fig. S7). Furthermore, of particular interest was the upregulation of growth promoting GLI2 targets including *Ccnd1*, *N-Myc*, *Akt1*, *Bcl2*, *Xiap*, and *Cdk2* (Fig. 3A). We validated the upregulation of these targets through qPCR using mouse pancreas tissue from all GEMM cohorts. Most target genes showed increased levels in KCRG mice compared with KC animals. However, only *Ccnd1*, *N-Myc*, and *Akt1* have a statistically significant increase in their expression in the KCRG cohort compared with the KC, as well as Cre and CRG controls (Fig. 3B; Supplementary Fig. S8).

Given the significant upregulation of *Ccnd1* (Fig. 3A) and its known role in PDAC biology (46–50), we used CCND1 as model for subsequent mechanistic analysis. Western blot analysis in the 1012U cells demonstrated an increase in CCDN1 protein expression when KRAS is induced post-doxycycline treatment (Fig. 3C). Further analysis showed that at both the mRNA and protein levels of CCDN1 in cells knockdown for *Gli2* (Fig. 3D and E). Additionally, we demonstrate that the overexpression of *Gli2* or *∆NGli2* increased the expression of CCND1 protein compared with empty vector control (Fig. 3F). Lastly, utilizing primers (Supplementary Table S3) flanking the candidate GLI binding site 1.8 kb upstream of *Ccnd1* transcriptional start site (TSS), we demonstrate GLI2 binding in the *Ccnd1* mouse promoter in the +Dox condition (Fig. 3G).

### GLI2 drives H3K4me3 enrichment downstream of oncogenic KRAS

**Figure 4:**
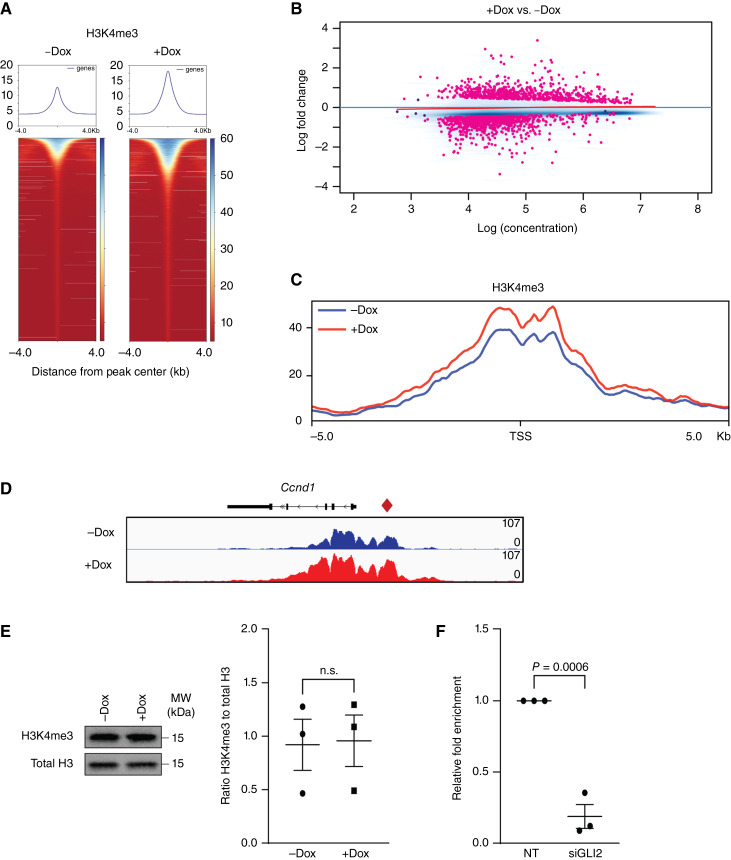
Gli2 drives H3K4me3 enrichment at Ccnd1 promoter in mutant KRAS cells. **A,** Heatmaps representing global levels of H3K4me3 enrichment in 1012U −Dox and 1012U +Dox cells. **B,** MA-plot highlighting differentially enriched H3K4me3 peaks in the 1012U +Dox cells compared with the 1012U −Dox cells. **C,** Profile plot of H3K4me3 at the transcriptional start site + or − 5 kb comparing 1012U +Dox cells with the 1012U −Dox cells. **D,** ChIP-seq tracks showing enrichment of H3K4me3 in 1012U –Dox and +Dox cells for GLI target gene *Ccnd1*. **E,** Western blot (left) and protein quantification (right) representing expression of H3K4me3 in 1012U cells +Dox. Total H3 is used as the loading control. **F,** H3K4me3 enrichment at *Ccdn1* promoter in 1012U +Dox cells postknockdown of Gli2.

Next, we aimed to investigate the mechanisms underlying this regulation of gene expression by the KRAS–GLI2 axis. We therefore performed ChIP-seq for three major histone active marks, (H3K4me1, H3K4me3, and H3K27Ac in our inducible cells at 72 hours post-doxycycline treatment. First, ChIP-seq results revealed global reorganization in the +Dox condition in all three histone markers (Fig. 4A and B; Supplementary Figs. S9A and S9B, S10A). Notably, the most significant changes are seen in H3K4me3 with differential enrichment analysis identifying 496 enriched promoter sites unique to the +Dox condition (Fig. 4B). In addition, integrative of analysis of global H3K4me3 and H3K27Ac demonstrated a correlation in the enrichment of both marks under oncogenic *Kras* (Supplementary Fig. S9B) in particular at GLI2 target genes promoters (Fig. 4C and D; Supplementary Fig. S9C and S9D). Of note, no changes in H3K4me1 were seen at the promoters of these targets (Supplementary Fig. S10C and S10D). Globally, we confirmed that the observed enrichment at target genes was not due to an increased level of the marks, as protein levels did not show differences between the − and +Dox conditions (Fig. 4E; Supplementary Figs. S9E and S10B). ChIP-PCR was conducted in cells knockdown for GLI2 resulted in a loss of H3K4me3 within the *Ccnd1* promoter (Fig. 4F). Taken together, these epigenomic and transcriptomic analyses establish that GLI2, downstream of oncogenic KRAS, facilitates the enrichment of H3K4me3 mark at the promoter region of GLI target gene including *Ccnd1*, to drive gene expression.

## Discussion

This report provides evidence of a novel mechanism of gene expression regulation in pancreatic cancer downstream of oncogenic KRAS, with GLI2 as an effector. Through our findings, we demonstrate that the oncogenic KRAS increases GLI target gene expression. Furthermore, this KRAS–GLI2 axis drives enrichment of H3K4me3 at GLI target genes like *Ccnd1*, and loss of GLI2 reduces H3K4me3 enrichment at the promoter of this proliferative gene. This study uncovers a novel mechanism regulating KRAS-induced gene expression in PDAC, through which the chromatin landscape is altered by the enrichment of activating H3K4me3 mark at GLI target genes that are associated with proliferation, thus potentially promoting disease severity and progression. A recent study elucidated the chromatin remodeling that occurs in response to oncogenic KRAS signaling in PDAC cells (51). In particular, KRAS signaling triggers the formation and activation of enhancers, super enhancers, and promoters (and differential enrichment of H3K27Ac, H3K4me1, and H3K4me3, respectively) to regulate gene expression. Changes in heterochromatin-associated marks (H3K9me3 and H3K27me3) had no significant impact on the transcriptome (51). Our study also demonstrates significant alterations in the chromatin landscape, particularly in global enrichment of promoter marks like H3K4me3 when the KRAS–GLI2 axis is upregulated. One potential mechanism that could be driving the upregulation of this KRAS–GLI2 axis may involve the family of epigenetic regulators histone–lysine N-methyltransferases 2 (KMT2). KMT2s catalyze the methylation of histone H3 lysine 4 (H3K4; ref. 52). KMT2s are often dysregulated in pancreatic cancer and are associated with poor patient survival (53, 54). Recruitment of KMT2s by GLI2 may be the underlying molecular event regulating this KRAS-induced gene expression. Candidate KMTs mediating this GLI2-driven gene transcription are KMT2F and KMT2G and to a lesser extent KMT2A and KMT2B. It has been demonstrated that KMT2F and KMT2G are recruited to promoters by oncogenic transcription factors (55) and have been shown to have H3K4me3 activity (55–57). KMT2A and KMT2B have reported to be present at promoters (58, 59), but there are also significant sources of evidence demonstrating the recruitment of these KMTs to enhancer elements (60, 61). Future studies will include elucidating how KMT2s participate in this KRAS–GLI2 axis and their impact in pancreatic carcinogenesis.

## Supplementary Material

Supplementary Figure 1 Supplementary Figure 1 shows correlation of SNP rs1992901 and GLI2 Transcript Expression.

Supplementary Figure 2 Supplementary Figure 2 describes the characterization of the impact Gli2 overexpression in pancreas development. showing that Gli2 loss has no impact on pancreas development or survival in vivo.

Supplementary Figure 3 Supplementary Figure 3 shows Gli2 expression in CRG and KCRG mice and Gli luciferase activity in ΔNGli2-transfected cells.

Supplementary Figure 4 Supplementary Figure 4 shows the validation of Kras signaling activation and chronic pancreatitis phenotypic examples in KC and KCRG mice.

Supplementary Figure 5 Supplementary Figure 5 shows the IHC results looking at CD4 and CD8 expression in KC and KCRG mice. The results show no difference in the immune landscape between these mouse models.

Supplementary Figure 6 Supplementary Figure 6 describes RNA-seq shows differential gene expression induced by oncogenic KRAS.

Supplementary Figure 7 Supplementary Figure 7 shows how oncogenic KRAS modulates GLI target gene expression.

Supplementary Figure 8 Supplementary Figure 8 shows the expression of GLI target genes in all experimental groups.

Supplementary Figure 9 Supplementary Figure 9 shows that Gli2 does not change H3K27Ac enrichment at Ccnd1 promoter downstream of oncogenic KRAS.

Supplementary Figure 10 Supplementary Figure 10 shows not differences in H3K4me1 enrichment at Ccnd1 promoter in mutant KRAS cells.

Supplementary Table 1 Supplementary Table 1 describes the characteristics of the patients used in our study.

Supplementary Table 2 Supplementary Table 2 includes the SNP analysis of components of the Hedgehog pathway.

Supplementary Table 3 Supplementary Table 3 includes the sequence of the primers used in our study.
