# Supplementary material for: KRAS Promotes GLI2-Dependent Transcription during Pancreatic Carcinogenesis
Source: Cancer Res Commun. 2024 Jul 9;4(7):1677–89. doi: 10.1158/2767-9764.CRC-23-0464 (PMC11232480; doi:10.1158/2767-9764.CRC-23-0464)
Supplement: Supplementary Figure 1 — shows correlation of SNP rs1992901 and GLI2 Transcript Expression. [file crc-23-0464_supplementary_figure_1_supp1.pdf]

A

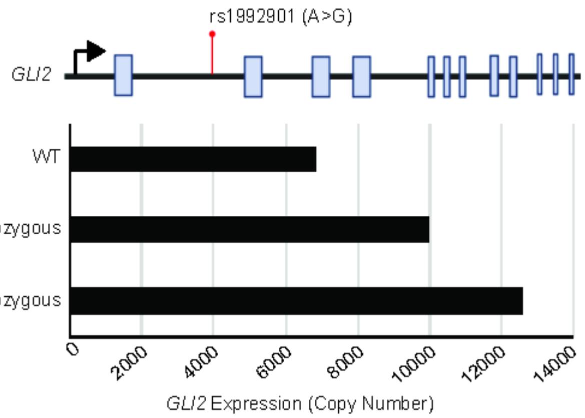

B

Gene	rs1992901	N	Median	Mean	p-value
SHH	WT	84	433.44	621.22	0.0621
	Heterozygous	82	258.37	552.16	
	Homozygous	25	572.01	828.05	
GLI1	WT	84	450.46	657.29	0.1921
	Heterozygous	82	554.79	653.06	
	Homozygous	25	614.06	866.67	
GLI2	WT	84	3695.29	6828.14	0.0142
	Heterozygous	82	3037.96	9985.38	
	Homozygous	25	8735.84	12582.17	

Supplementary Figure S1: GLI2 SNP alters GLI2 rs1992901 Transcript Expression.
A. Upper: Graphical representation of *GLI2* gene with localization of SNP rs1992901. Lower: Graphical representation of mean GLI2 expression in patient samples with wildtype (WT) (n=84), heterozygous (n=82) and homozygous (n=25) GLI2 SNP rs1992901. B. SHH, GLI1 and GLI2 gene expression in patient samples with WT, heterozygous or homozygous GLI2 SNP rs1992901.
