# Supplementary material for: KRAS Promotes GLI2-Dependent Transcription during Pancreatic Carcinogenesis
Source: Cancer Res Commun. 2024 Jul 9;4(7):1677–89. doi: 10.1158/2767-9764.CRC-23-0464 (PMC11232480; doi:10.1158/2767-9764.CRC-23-0464)
Supplement: Supplementary Figure 2 — describes the characterization of the impact Gli2 overexpression in pancreas development. showing that Gli2 loss has no impact on pancreas development or survival in vivo. [file crc-23-0464_supplementary_figure_2_supp2.pdf]

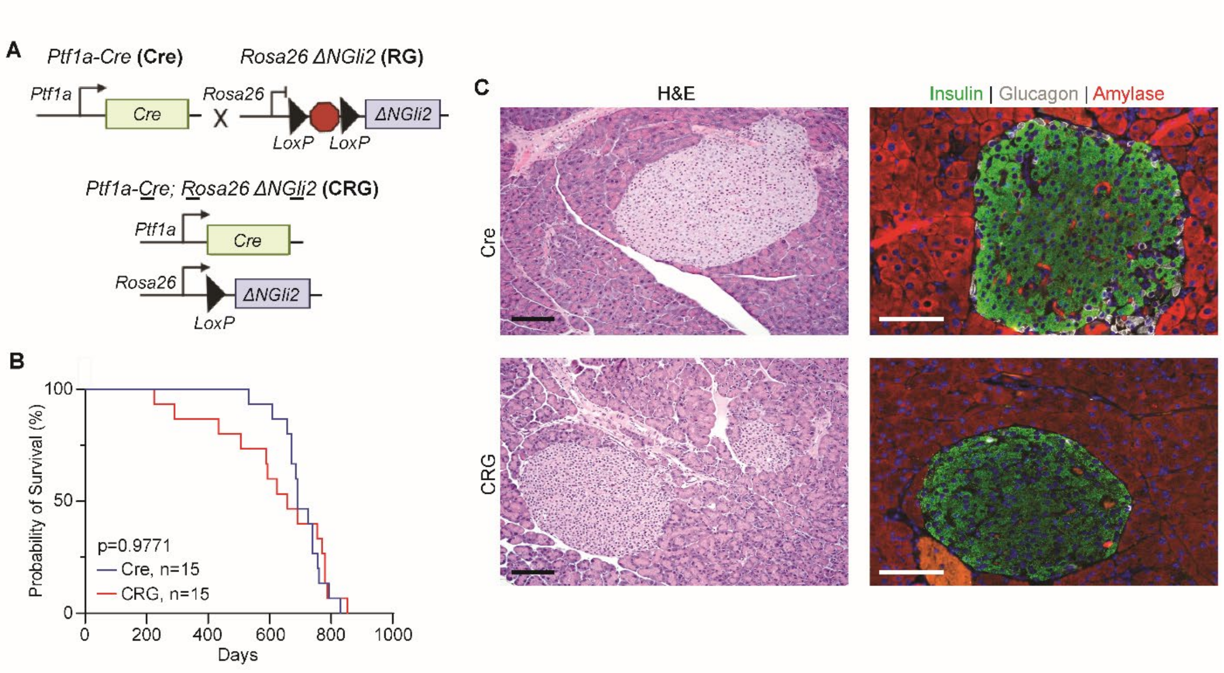

Supplementary Figure S2: Gli2 loss has no impact on pancreas development or survival *in vivo*.

A. Representation of animal cross to generate *Ptf1a-Cre; Rosa26 ΔNGli2* (CRG) mice from *Ptf1a-Cre* (Cre) and *Rosa26 ΔNGli2* (RG) mice. B. Kaplan-Meier curve representing overall survival in Cre (n=15) and CRG (n=15) mice (Log-Rank (Mantel-Cox) test, p=0.9771). C. Left panel: Representative H&E stain images in Cre and CRG mice showing pancreas tissue morphology. Scale bar: 100 µm. Right panel: Immunofluorescence stain for insulin (green), glucagon (white) and amylase (red) in pancreas tissue sections from Cre and CRG mice. Scale bar: 50 µm.
