# Supplementary material for: KRAS Promotes GLI2-Dependent Transcription during Pancreatic Carcinogenesis
Source: Cancer Res Commun. 2024 Jul 9;4(7):1677–89. doi: 10.1158/2767-9764.CRC-23-0464 (PMC11232480; doi:10.1158/2767-9764.CRC-23-0464)
Supplement: Supplementary Figure 3 — shows Gli2 expression in CRG and KCRG mice and Gli luciferase activity in ΔNGli2-transfected cells. [file crc-23-0464_supplementary_figure_3_supp3.pdf]

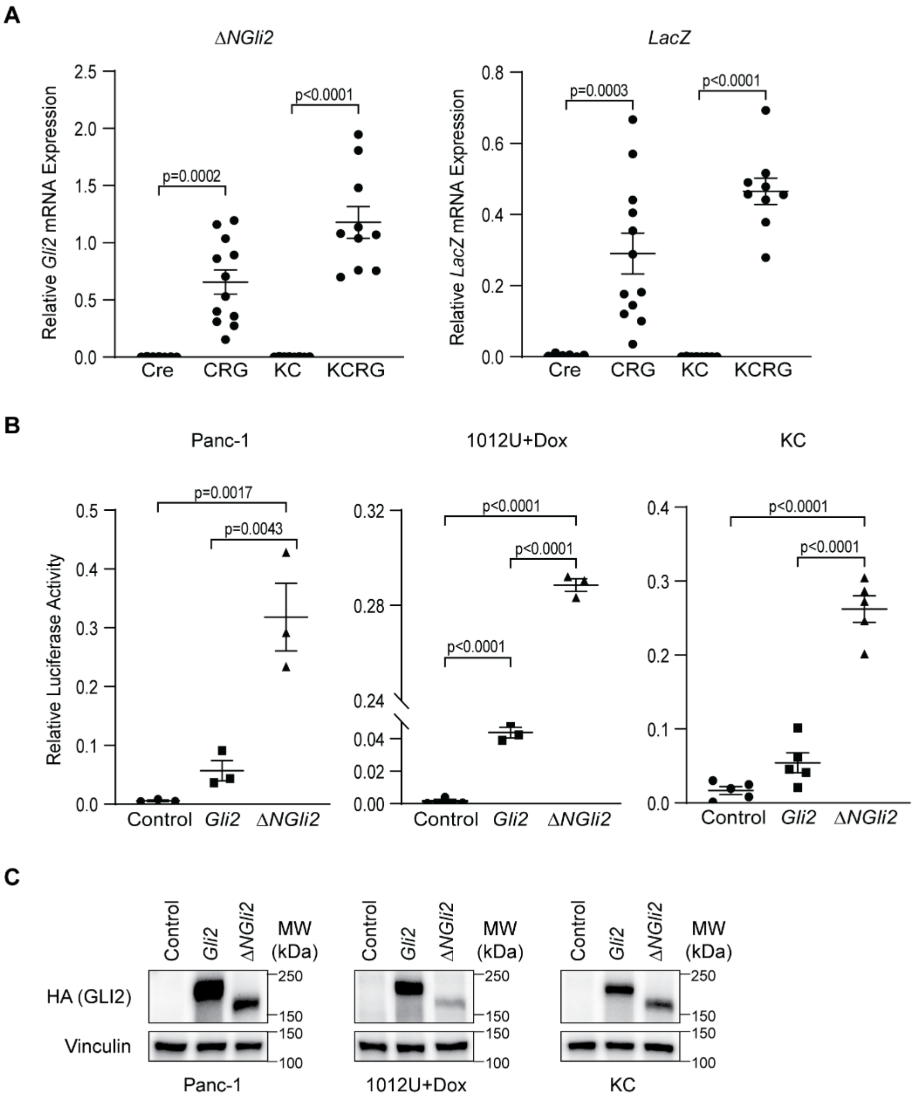

Supplementary Figure S3: Gli2 expression is confirmed in CRG and KCRG mice and Gli luciferase activity was increased in ΔNGli2-transfected cells.

A. Left panel: Gene expression of *Gli2* through qPCR to validate recombination of *Rosa26 ΔNGli2* in CRG (p=0.0002) and KCRG (p<0.0001) mouse pancreas tissue. Right panel: Gene expression of *LacZ* through qPCR to validate recombination of *Rosa26 ΔNGli2* in CRG (p=0.0003) and KCRG (p<0.0001) mouse pancreas tissue. B. 8xGLI luciferase reporter assay in Panc-1, 1012U +Dox and murine KC cell line transfected with *Gli2*, *ΔNGli2* or empty vector (control). C. Western blot representing expression of GLI2 (HA-tagged) in Panc-1, 1012U +Dox and murine KC cell line transfected with *Gli2*, *ΔNGli2* or empty vector (control). Vinculin is used as loading control.
