# Supplementary material for: KRAS Promotes GLI2-Dependent Transcription during Pancreatic Carcinogenesis
Source: Cancer Res Commun. 2024 Jul 9;4(7):1677–89. doi: 10.1158/2767-9764.CRC-23-0464 (PMC11232480; doi:10.1158/2767-9764.CRC-23-0464)
Supplement: Supplementary Figure 4 — shows the validation of Kras signaling activation and chronic pancreatitis phenotypic examples in KC and KCRG mice. [file crc-23-0464_supplementary_figure_4_supp4.pdf]

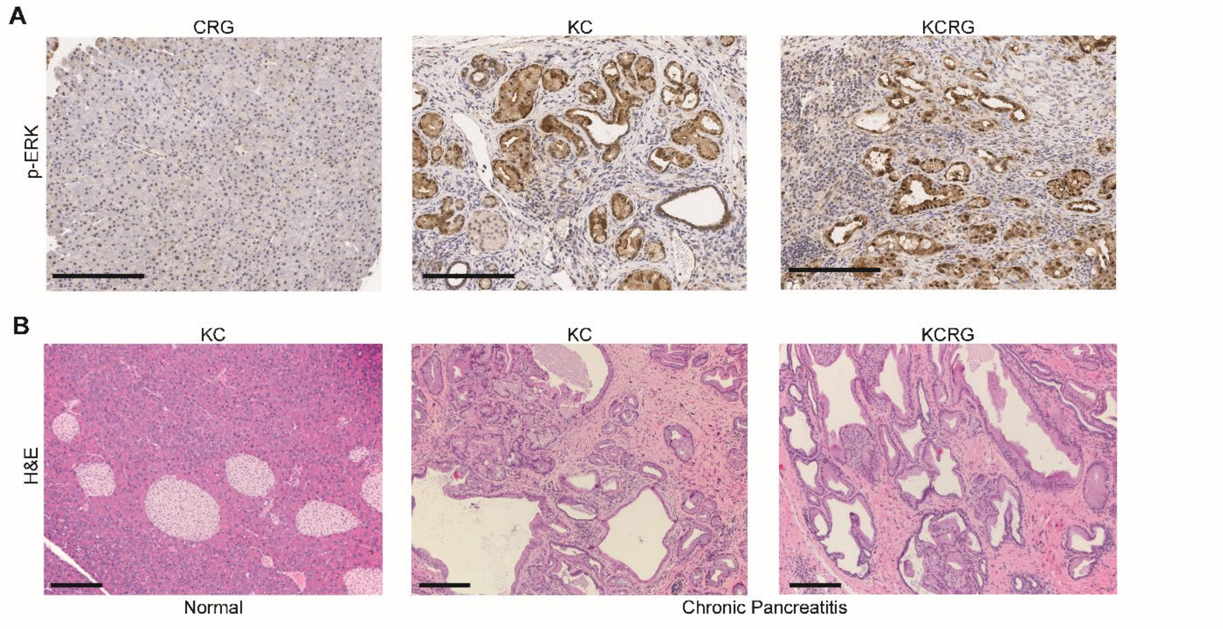

Supplementary Figure S4: Validation of Kras signaling activation and chronic pancreatitis histology in KC and KCRG mice.

A. Representative IHC images for p-ERK (brown signal) in CRG, KC and KCRG mice verifying Kras signal activation in mice from KC and KCRG cohorts. Scale bar: 200 µm. B. H&E stain in KC and KCRG mice representative of normal histology and chronic pancreatitis. Scale bar: 100 µm.
