# Supplementary material for: KRAS Promotes GLI2-Dependent Transcription during Pancreatic Carcinogenesis
Source: Cancer Res Commun. 2024 Jul 9;4(7):1677–89. doi: 10.1158/2767-9764.CRC-23-0464 (PMC11232480; doi:10.1158/2767-9764.CRC-23-0464)
Supplement: Supplementary Figure 5 — shows the IHC results looking at CD4 and CD8 expression in KC and KCRG mice. The results show no difference in the immune landscape between these mouse models. [file crc-23-0464_supplementary_figure_5_supp5.pdf]

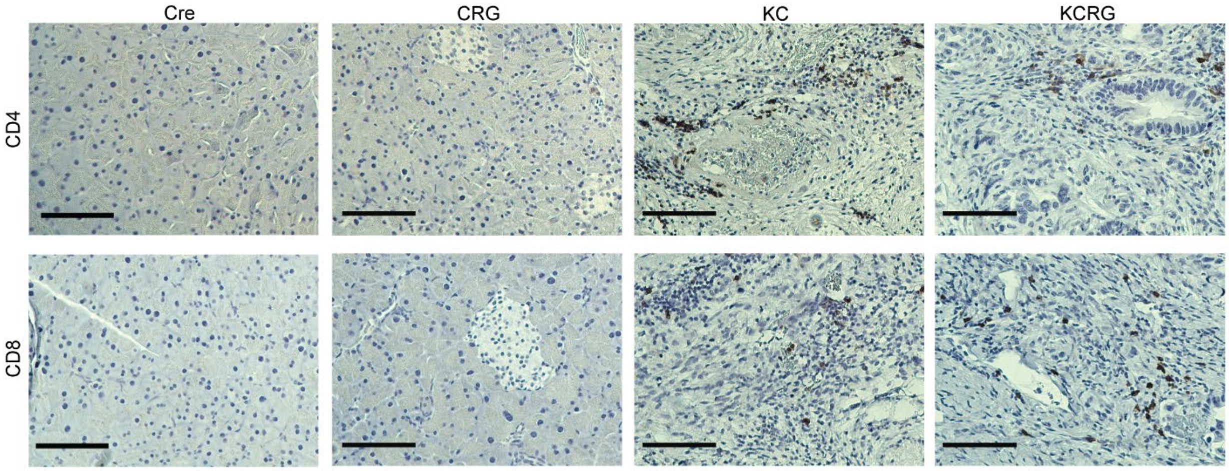

Supplementary Figure S5: KC and KCRG showed no difference in the immune landscape.

Representative IHC images for CD4, and CD8 (brown signal) in Cre, CRG, KC and KCRG mice showing the distribution of immune cells in these cohorts. Scale bar: 200 µm.
