# Supplementary material for: KRAS Promotes GLI2-Dependent Transcription during Pancreatic Carcinogenesis
Source: Cancer Res Commun. 2024 Jul 9;4(7):1677–89. doi: 10.1158/2767-9764.CRC-23-0464 (PMC11232480; doi:10.1158/2767-9764.CRC-23-0464)
Supplement: Supplementary Figure 6 — describes RNA-seq shows differential gene expression induced by oncogenic KRAS. [file crc-23-0464_supplementary_figure_6_supp6.pdf]

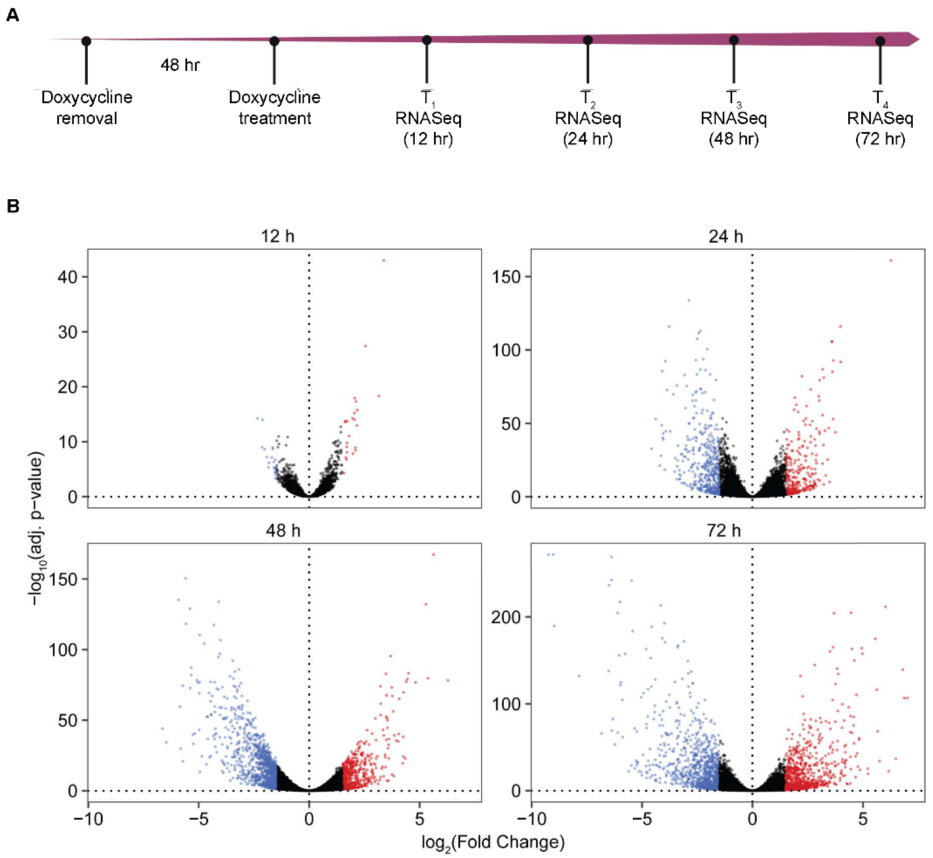

Supplementary Figure S6: Oncogenic KRAS induces differential gene expression.
A. Schematic representing timeline of doxycycline treatment of 1012U cells and sample extraction for RNA-seq and ChIP-seq. B. Volcano plots representing differentially expressed genes (DEGs) in 1012U +Dox cells at 12, 24, 48 and 72 h post doxycycline treatment.
