# Supplementary material for: KRAS Promotes GLI2-Dependent Transcription during Pancreatic Carcinogenesis
Source: Cancer Res Commun. 2024 Jul 9;4(7):1677–89. doi: 10.1158/2767-9764.CRC-23-0464 (PMC11232480; doi:10.1158/2767-9764.CRC-23-0464)
Supplement: Supplementary Figure 7 — shows how oncogenic KRAS modulates GLI target gene expression. [file crc-23-0464_supplementary_figure_7_supp7.pdf]

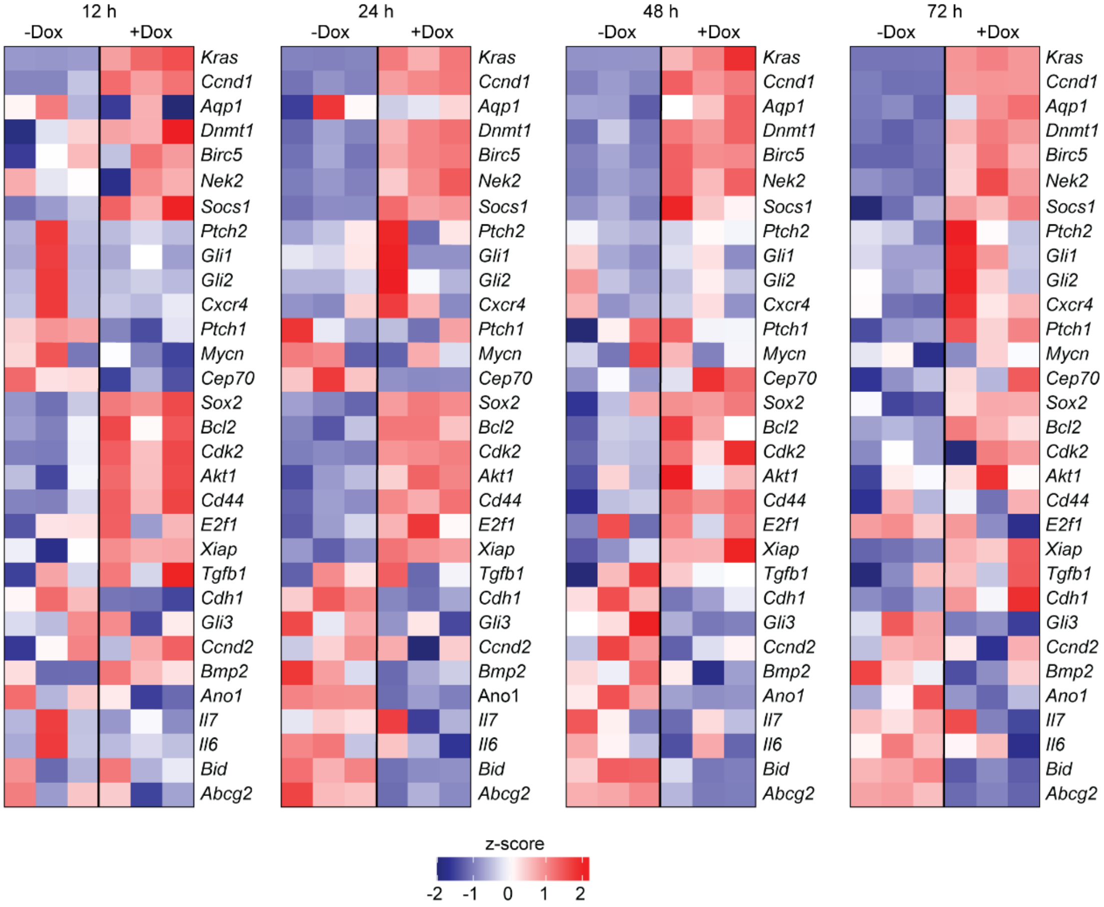

Supplementary Figure S7: Oncogenic KRAS modulates GLI target gene expression. Heatmaps representing GLI target gene expression in 1012U –Dox and +Dox cells at 12, 24, 48 and 72 h doxycycline treatment.
