# Supplementary material for: KRAS Promotes GLI2-Dependent Transcription during Pancreatic Carcinogenesis
Source: Cancer Res Commun. 2024 Jul 9;4(7):1677–89. doi: 10.1158/2767-9764.CRC-23-0464 (PMC11232480; doi:10.1158/2767-9764.CRC-23-0464)
Supplement: Supplementary Figure 8 — shows the expression of GLI target genes in all experimental groups. [file crc-23-0464_supplementary_figure_8_supp8.pdf]

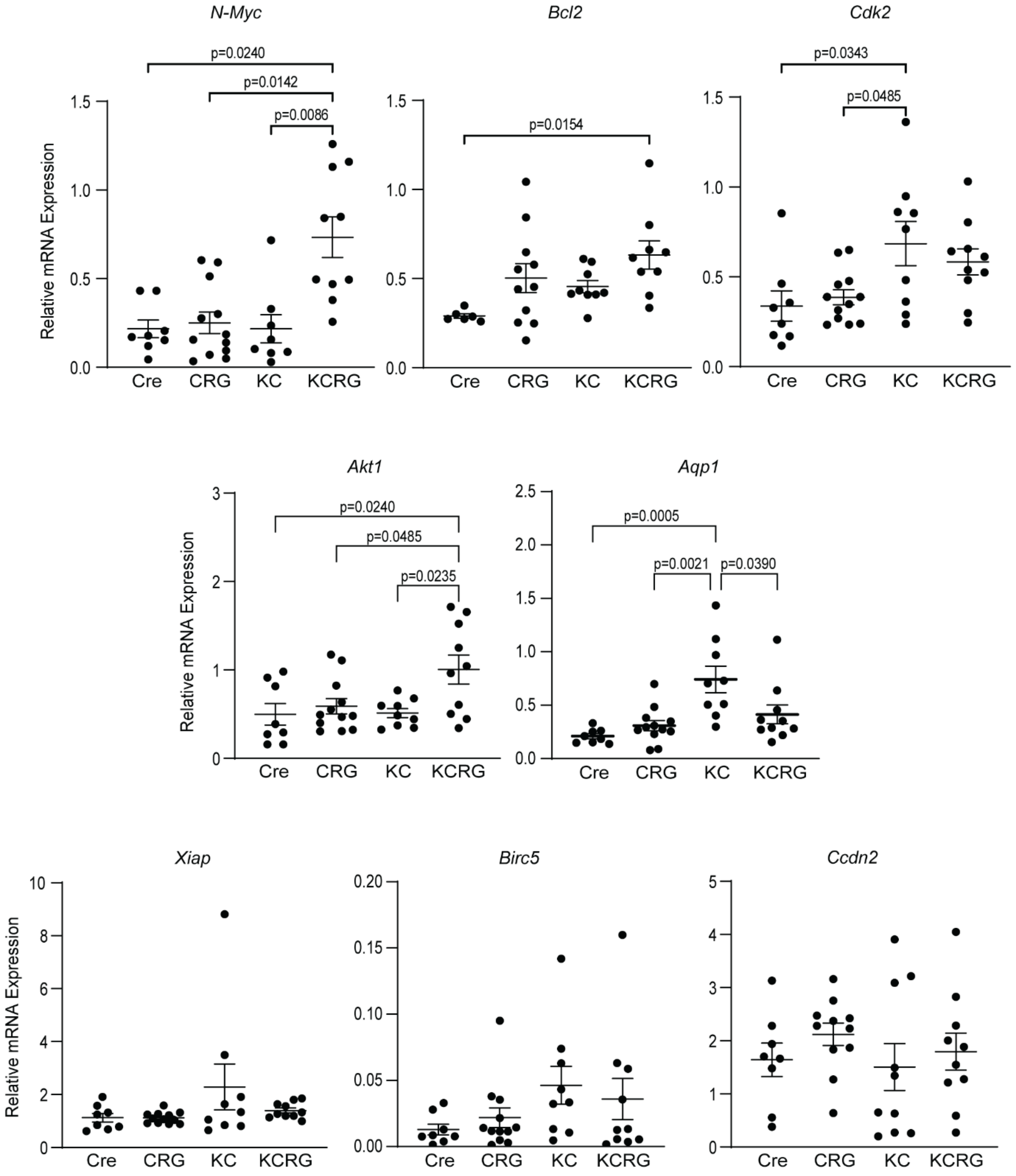


**Supplementary Figure S8: Expression of GLI target genes in all experimental groups.** Gene expression of GLI targets *N-Myc*, *Bcl2*, *Cdk2*, *Akt1*, *Aqp1*, *Xiap*, *Birc5* and *Ccdn2* using qPCR in mouse pancreas tissue from Cre, CRG, KC and KCRG mice.
