# Supplementary material for: KRAS Promotes GLI2-Dependent Transcription during Pancreatic Carcinogenesis
Source: Cancer Res Commun. 2024 Jul 9;4(7):1677–89. doi: 10.1158/2767-9764.CRC-23-0464 (PMC11232480; doi:10.1158/2767-9764.CRC-23-0464)
Supplement: Supplementary Figure 10 — shows not differences in H3K4me1 enrichment at Ccnd1 promoter in mutant KRAS cells. [file crc-23-0464_supplementary_figure_10_supp10.pdf]

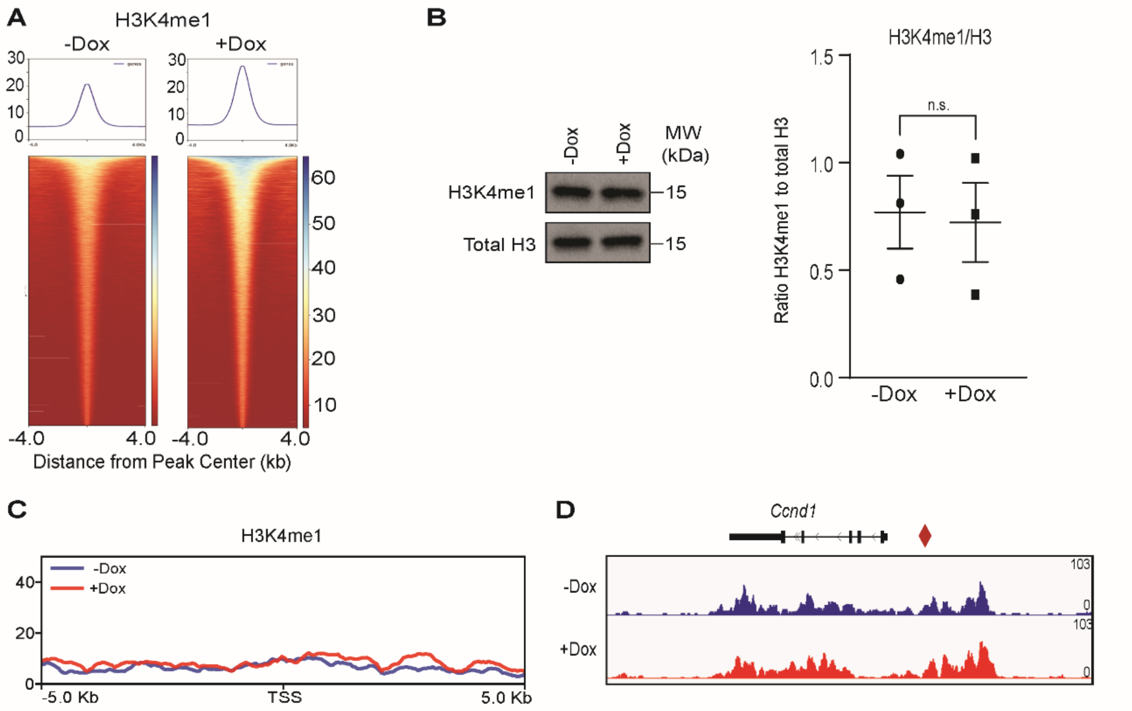

Supplementary Figure S10: H3K4me1 enrichment at Ccnd1 promoter was not altered by mutant KRAS.

A. Heatmaps representing global levels of H3K4me1 enrichment in 1012U –Dox and 1012U +Dox cells. B. Western blot (left panel) and protein quantification (right panel) representing expression of H3K4me1 in 1012U cells + Dox. Total H3 mark is used as loading control. C. Profile plot of H3K4me1 at the transcriptional start site + or - 5kb comparing 1012U +Dox cells to the 1012U –Dox cells. D. ChIP-seq tracks showing enrichment of H3K4me1 marks in 1012U –Dox and +Dox cells for GLI target gene *Ccnd1*.
